# Supplementary material for: Predictive computational phenotyping and biomarker discovery using reference-free genome comparisons
Source: BMC Genomics. 2016 Sep 26;17:754. doi: 10.1186/s12864-016-2889-6 (PMC5037627; doi:10.1186/s12864-016-2889-6)
Supplement: Additional file 1 — Appendix. The appendix contains supporting information and detailed results that are not essential to the reader’s understanding of the key findings of this study. (PDF 258 kb) [file 12864_2016_2889_MOESM1_ESM.pdf]

Predictive computational phenotyping and biomarker discovery using reference-free genome comparisons

Additional File 1 — Appendix

Alexandre Drouin*, Sébastien Giguère, Maxime Déraspe, Mario Marchand, Michael Tyers, Vivian G Loo, Anne-Marie Bourgault, François Laviolette†, Jacques Corbeil†

Appendix 1 The Set Covering Machine

The Set Covering Machine (SCM) [1], is a learning algorithm that produces models that are conjunctions (logical-AND) or disjunctions (logical-OR) of boolean-valued rules $r : \mathbb{R}^d \rightarrow \{0, 1\}$. Let us use $h(\mathbf{x})$ to denote the output of model h on genome $\mathbf{x}$. When h consists of a conjunction (i.e., a logical-AND) of a set $\mathcal{R}^\star$ of rules $r^\star$, we have

$$h(\mathbf{x}) = \bigwedge_{r^\star \in \mathcal{R}^\star} r^\star(\mathbf{x})\,. \tag{1}$$

Whereas, for a disjunction (i.e., a logical-OR) of rules, we have

$$h(\mathbf{x}) = \bigvee_{r^\star \in \mathcal{R}^\star} r^\star(\mathbf{x})\,. \tag{2}$$

Given a set $\mathcal{R}$ of candidate rules, the SCM attempts to find a model that minimizes the empirical error rate $R_\mathcal{S} \stackrel{\text{def}}{=} \frac{1}{m}\sum_{i=1}^{m} I[h(\mathbf{x}_i) \neq y_i]$, where $I[\text{True}] = 1$ and 0 otherwise, while using the smallest number of rule in $\mathcal{R}$. This problem reduces to the minimum set cover problem, which is known to be NP-hard [1, 2]. To overcome this issue, the SCM uses a greedy optimisation algorithm inspired by the greedy set cover algorithm [3], which yields an approximate solution with a good worst case guarantee. In addition, there is empirical evidence that the solution found using the greedy heuristic is very close to optimality [4] .

Algorithm 1 presents the SCM algorithm for the case where the returned model is a conjunction of boolean-valued rules. For the sake of conciseness, we only present the conjunction case. Let $\neg b$ denote the boolean complement of b. The disjunction case can be obtained from Algorithm 1 by using $\mathcal{S}' = \{(\mathbf{x}_i, \neg y_i) : (\mathbf{x}_i, y_i) \in \mathcal{S}\}$ as the set of training examples and by taking the complement of the returned model, $\neg h$ as the model. This follows from the De Morgan law: $\neg(\bigwedge_{r^\star \in \mathcal{R}^\star} r^\star(\mathbf{x})) = \bigvee_{r^\star \in \mathcal{R}^\star} \neg r^\star(\mathbf{x})$.

The SCM starts with an empty conjunction and extends it in a greedy manner by iteratively selecting the rule maximizing a utility function. The latter is designed to favor conjunctions that correctly classify most of the training examples, while taking into account some constraints imposed by a greedy approach. Since the algorithm is greedy, once a rule is added to the conjunction, it cannot be removed. Observe that, any rule in the conjunction (logical-AND) that assigns the negative class to an example forces the result of

*Correspondence: alexandre.drouin.8@ulaval.ca
†Shared senior authorship

the conjunction itself to be negative. Therefore, there are two types of errors to consider: errors on negative examples, which can be recovered, and errors on positive examples, which are definitive. For this reason, Marchand and Shawe-Taylor [1] have proposed to score each rule using the following utility function:

$$U \stackrel{\text{def}}{=} |\mathcal{A}| - p \cdot |\mathcal{B}|, \tag{3}$$

where $|\mathcal{A}|$ is the number of negative examples that it correctly classifies (i.e., covered by that rule) and $|\mathcal{B}|$ is the number of positive examples on which it errs. A hyperparameter p, that is usually selected by cross-validation, allows to fix the correct trade-off between these two types of errors.

At each iteration, examples that are classified as negative by the selected rule are excluded from further evaluations of the utility function. Indeed, for these examples, the result of the conjunction will necessarily remain negative. There is therefore no need to consider them while extending the model. This also ensures that redundant rules are not added to the conjunction, effectively favoring sparse models. This iterative process is repeated until one of the three following stopping criterion is reached.

The first stopping criterion is reached when all the negative examples are correctly classified by the rules of the conjunction. In this case, there is no need to continue extending the conjunction, since adding rules can only increase the number of errors on the positive examples. The second stopping criterion is reached when the number of rules in the conjunction reaches a pre-defined limit s. This limit is a hyperparameter that induces regularization by early-stopping. Finally, the third stopping criterion is reached when the rule of maximal utility has $|\mathcal{A}| = 0$ and $|\mathcal{B}| = 0$. In this case, the algorithm is in a state of equilibrium, since no examples are removed at the end of the iteration.

Note that the version of the SCM presented here differs in two points from the one of Marchand and Shawe-Taylor [1]. First, when more than one rule have the maximal utility, it selects the rule with the smallest empirical error rate. This simple strategy is important for genomic datasets where the number of features is much greater than the number of examples. It becomes particularly important after a few iterations, as fewer examples contribute to the utility function and a lot of rules may have the same utility. In this situation, it is reasonable to assume that the rule that has the best performance on all the examples of the training set, is more likely to contribute to the best generalization performance. Second, the algorithm is stopped when it reaches the state of equilibrium mentioned above. This prevents from adding useless rules and reduces the training time.

The worst-case running time complexity of Algorithm 1 is $O(|\mathcal{R}| \cdot |\mathcal{S}| \cdot s)$. It thus scales linearly in the number of rules and the number of training examples. This is a noteworthy characteristic of the SCM algorithm, as it is essential for scaling to large genomic datasets.

Algorithm 1 Set Covering Machine (Conjunction)

Input: $\mathcal{S}$: A set of m training examples, $\mathcal{R}$: A set of boolean-valued rules, p: The trade-off parameter, s: The early stopping parameter

$\mathcal{R}^\star \leftarrow \emptyset$

$\mathcal{P} \leftarrow$ the set of examples in $\mathcal{S}$ with label 1

$\mathcal{N} \leftarrow$ the set of examples in $\mathcal{S}$ with label 0

$stop \leftarrow False$

while $\mathcal{N} \neq \emptyset$ **and** $|\mathcal{R}^\star| < s$ **and** $\neg stop$ **do**

  ▷ Compute the utility function for each rule

    $\forall i \in \{1, ..., |\mathcal{R}|\}$,

      $\mathcal{A}_i \leftarrow$ the subset of $\mathcal{N}$ correctly classified by r_i

      $\mathcal{B}_i \leftarrow$ the subset of $\mathcal{P}$ misclassified by r_i

      $U_i \leftarrow |\mathcal{A}_i| - p \cdot |\mathcal{B}_i|$

  ▷ Select the best rule

    $U^\star \leftarrow \max\limits_{i \in \{1,...,|\mathcal{R}|\}} U_i$

    $\mathcal{C} \leftarrow \{i \in \{1, ..., |\mathcal{R}|\} \mid U_i = U^\star\}$

    $i^\star \leftarrow \operatorname*{argmin}\limits_{i \in \mathcal{C}} \sum_{j=1}^{m} I[r_i(\mathbf{x}_j) \neq y_j]/m$

  ▷ Add the best rule to the conjunction

    if $|\mathcal{A}_{i^\star}| > 0$ **or** $|\mathcal{B}_{i^\star}| > 0$ **then**

      $\mathcal{R}^\star \leftarrow \mathcal{R}^\star \cup \{r_{i^\star}\}$

      $\mathcal{N} \leftarrow \mathcal{N} - \mathcal{A}_{i^\star}$

      $\mathcal{P} \leftarrow \mathcal{P} - \mathcal{B}_{i^\star}$

    else

      $stop \leftarrow True$

    endif

end while

return h, where $h(\mathbf{x}) = \bigwedge_{r^\star \in \mathcal{R}^\star} r^\star(\mathbf{x})$

Appendix 2 Removing genes from the data

This section describes the protocol that was used to remove the k-mers associated with a specific gene from the data (e.g.: *pbp1a*, *pbp2b*). First, the amino acid sequence corresponding to the wild-type version of the gene was acquired from UniProt. Then, in order to find occurrences of the gene in the genomes of the dataset, we performed a tfastx alignment [5] over all the contigs of each genome. We considered that a sequence was an occurrence of the gene if it had more than 90% identity with the wild-type sequence. In our experiments, this yielded a single occurrence per genome. Then, all the identified gene sequences were k-merized and the resulting k-mers were removed from the dataset. Hence, we filtered the k-mers belonging to each variant of the gene that was present the dataset.

Appendix 3 The k-mer length as a hyperparameter

In the absence of prior knowledge for setting the k-mer length, we propose to consider k as a hyperparameter of the algorithm. Its value can then be selected by cross-validation or, as described in Appendix 4, by using the sample-compression bound. This method was evaluated by repeating the experiments presented in Section *Results – The SCM models are sparse and accurate* of the main manuscript, while using cross-validation to select $k \in \{15, 21, 31, 51, 71, 91\}$.

The results, which are shown in Table A1, indicate that setting the k-mer length by cross-validation, instead of using $k = 31$, yields similar results in terms of error rate ($p = 0.551$) and sparsity ($p = 0.783$). This corroborates that k-mers of length 31 are well-suited for bacterial genome comparisons. It also indicates that cross-validation can recover a good k-mer length in the absence of prior knowledge.

Table A1: Comparison of a SCM that uses k-mers of length 31 (SCM-31) and a SCM that uses cross-validation to select the k-mer length. The values in the table are averages over 10 repetitions of the experiment. For each dataset and method, the error rate and the number of k-mers in the model (in parentheses) are shown. The average k-mer length selected by cross-validation is also shown along with the standard deviation. The best error rates are in bold.

Dataset	SCM-31	SCM-CV	k-mer length
C. difficile			
Azithromycin	**0.030** (3.3)	0.032 (3.2)	40.0 ± 23.0
Ceftriaxone	**0.073** (2.6)	0.085 (3.0)	41.0 ± 31.3
Clarithromycin	**0.011** (3.0)	0.018 (2.9)	26.0 ± 12.0
Clindamycin	0.021 (1.4)	**0.011** (1.9)	56.0 ± 21.6
Moxifloxacin	**0.020** (1.0)	**0.020** (1.0)	39.0 ± 16.0
M. tuberculosis			
Ethambutol	0.179 (1.4)	**0.164** (1.2)	49.0 ± 22.7
Isoniazid	**0.021** (1.0)	**0.021** (1.0)	24.0 ± 4.6
Pyrazinamide	**0.318** (3.1)	0.353 (2.3)	35.0 ± 24.6
Rifampicin	0.031 (1.4)	**0.006** (1.0)	49.0 ± 6.0
Streptomycin	0.050 (1.0)	**0.048** (1.0)	25.0 ± 4.9
P. aeruginosa			
Amikacin	**0.175** (4.9)	0.188 (4.9)	43.0 ± 28.2
Doripenem	**0.270** (1.4)	**0.270** (1.8)	39.0 ± 28.6
Levofloxacin	**0.072** (1.2)	0.073 (1.4)	34.0 ± 12.7
Meropenem	**0.267** (1.6)	0.276 (2.0)	32.0 ± 14.5
S. pneumoniae			
Benzylpenicillin	0.013 (1.1)	**0.011** (1.0)	50.0 ± 28.8
Erythromycin	**0.037** (2.0)	0.038 (1.9)	39.0 ± 29.6
Tetracycline	**0.031** (1.1)	**0.031** (1.1)	55.0 ± 24.6

In addition, various k-mer lengths were selected throughout the 10 repetitions of the experiment, but the results were found to be similar to those obtained with a fixed $k = 31$. This suggests that the k-mer length does not have a significant impact on the accuracy of the models. This is consistent with the sample-compression bound, which suggests that the generalization performance of the SCM is independent of the k-mer length, as long as it is

possible to find a model that makes few errors on the training data.

Finally, the existence of overlap between models generated from different k-mer lengths for a same antibiotic was investigated. It was observed that k-mer overlap does indeed occur frequently, since k-mers in highly conserved regions can be extended towards their $5'$ or $3'$ end until a mutation is observed in one of the training examples. However, there are cases, such as the one presented in Table A2, where overlap does not occur. In this example, extending the k-mer in the $k = 15$ model to length 21 leads to a model of lesser accuracy. This occurs due to perturbations in the genomic data, such as a mutation in a single training example. Hence, when learning the $k = 21$ model, the algorithm favored another locus. Interestingly, the difference in error rate between the $k = 15$ and $k = 21$ models is negligible and both k-mers map to the same region of the *P. aeruginosa* genome, 252 base pairs apart. Hence, despite the absence of sequence overlap, both k-mers are equivalently predictive biomarkers of the phenotype.

Table A2: An example, drawn from the results for the doripenem dataset (*P. aeruginosa*), where extending the k-mer in the best $k = 15$ model does not lead to the best model for $k = 21$.

Model	Rule	Error Rate
Best model ($k = 15$)	Presence(AAACCCTCGCGAAAT)	0.212
Best Superstring ($k = 21$)	Presence(AAACCCTCGCGAAATTTCGCA)	0.220
Best model ($k = 21$)	Presence(CATCCTCGATCCGGGGATGGG)	0.216

Appendix 4 An upper bound on the error rate

4.1 Derivation of the bound

The setting in which m, the number of example, is much smaller than d, the dimensionality of the feature space, is well known to be challenging for machine learning algorithms (see [6]). Indeed, in this context, it is common for learning algorithms to overfit the training data and thus, have a poor generalization performance. Genomic biomarker discovery fits precisely in this regime, since the datasets contain hundreds to thousands of genomes, while the genomes themselves contain several millions of features. Below, we present a counter-intuitive, yet essential, theoretical result, that shows that our approach can achieve good generalization performance in this challenging setting.

Based on the pioneering work of Littlestone and Warmuth [7], and Floyd and Warmuth [8], Marchand and Sokolova [9] performed a theoretical analysis of the generalization performance of the Set Covering Machine. They obtained an upper bound on the error rate of any model learned using this algorithm. Their bound is valid, under the condition that any model h considered by the SCM can be specified by two complimentary sources of information: a small subset $\mathcal{Z}$ of the training data (called the compression set) and a message string σ that contains the additional information needed to identify the model. In our case, the compression set $\mathcal{Z}$ for a model h is the smallest set of genomes of the training set in which there is at least one genome containing any k-mer associated to a rule in h. Moreover, σ is a message string that specifies: 1) the number $|h|$ of rules in model h, 2) the location, in base pairs, of the k-mer associated to each rule in h in the genomes of $\mathcal{Z}$ (only one occurrence), 3) the type (absence/presence) for each rule of h. Notice then that h can be fully reconstructed using the information contained in $\mathcal{Z}$ and σ.

To use the bound of [9], we must define a prior probability distribution $P_{\mathcal{Z}}(\sigma)$ over the possible values of σ, given a compression set $\mathcal{Z}$. This distribution must assign a prior probability to any combination of values for the three elements specified by σ. First, we attribute a probability of $\zeta(|h|) = (6/\pi^2)(|h|+1)^{-2}$ to each possible number $|h|$ of rules in the model. This value of ζ was chosen, since it slowly decreases with $|h|$ and $\sum_{|h|=0}^{\infty} \zeta(|h|) = 1$. Second, for each k-mer associated to a rule in h, we attribute a uniform probability of $1/|\mathcal{Z}|$, to every possible location in $\mathcal{Z}$, where $|\mathcal{Z}|$ denotes the total number of base pairs in $\mathcal{Z}$ (below, we use $m_{\mathcal{Z}}$ to denote the number of genomes in $\mathcal{Z}$). Third, each type of rule is given the same prior probability of 1/2. Hence, the prior is given by

$$P_{\mathcal{Z}}(\sigma) = \frac{6}{\pi^2}(|h|+1)^{-2}\left(\frac{1}{|\mathcal{Z}|}\cdot\frac{1}{2}\right)^{|h|} . \tag{4}$$

By inserting this prior distribution in the bound of [9], we obtain the following upper bound on the error rate $R(h)$ of any model h that can be learned using our proposed approach. More formally, with probability at least $1-\delta$ over all datasets $\mathcal{S}$ drawn according to D^m, we have that all models h have $R(h) \leq \epsilon$, where

$$\epsilon = 1 - \exp\left(\frac{-1}{m - m_{\mathcal{Z}} - r}\left[\ln\binom{m}{m_{\mathcal{Z}}} + \ln\binom{m - m_{\mathcal{Z}}}{r} + |h|\cdot\ln(2\cdot|\mathcal{Z}|) + \ln\left(\frac{\pi^6(|h|+1)^2(r+1)^2(m_{\mathcal{Z}}+1)^2}{216\cdot\delta}\right)\right]\right), \tag{5}$$

where r denotes the number of errors made by h on $\mathcal{S} \setminus \mathcal{Z}$.

Notice that this bound indicates good generalization for models that are sparse ($|h| \ll m$) and that have a small empirical error rate ($r \ll m$). This results is counter-intuitive, since the bound does not explicitly depend on the size k of k-mers used in the model. This is in sharp contrast with the more traditional theoretical analysis based on Occam's Razor [10], for which ϵ would grow linearly with $k \cdot |h|/\sqrt{m}$ – thus indicating a danger of overfitting whenever $k \cdot |h|$ is comparable to $\sqrt{m}$. The sample-compression analysis presented here implies that the model can use large k-mers and moderate sample sizes m without overfitting. Our proposed method is, therefore, particularly well suited for learning from genomic data.

4.2 Using the bound to replace cross-validation

The most common way of performing hyperparameter selection is by using cross-validation [6]. This method consists in splitting the dataset into c non-overlapping parts, called folds. One at a time, the folds are left out. The $c - 1$ remaining folds are used to train the learning algorithm. Then generalization performance of the obtained model is evaluated against the left out fold. A score, which corresponds to the average of the error rates achieved on each left out fold is assigned to each combination of hyperparameter values. Finally, the combination with the best score is selected and used to retrain the algorithm on the entire dataset in order to obtain a model. Notice that scoring a hyperparameter combination requires c trainings of the algorithm.

As an alternative to cross-validation, Marchand and Shawe-Taylor have proposed to score each combination of hyperparameters by using the value of an upper bound on the error rate that depends on the algorithm performance on the training set [1]. The hope is that, provided that this bound is tight enough, it could replace cross-validation in scoring the hyperparameter values, while requiring only a single training of the algorithm per hyperparameter combination. This would lead to an important reduction in the running time of experiments. By using their proposed bound, Marchand and Shawe-Taylor obtained results that are comparable to the ones obtained with cross-validation. However, they have only performed this analysis on datasets containing less than 20 features, which is far less than the ones that we consider.

We have attempted to use the sample-compression bound proposed in Equation (5) in order to select the values of the hyperparameters of the SCM. For each combination of hyperparameter values, we trained the algorithm using the entire training set $\mathcal{S}$, which yielded a model h. Then, the values required to compute the bound were obtained as follows. The values of m and $|h|$ are trivially obtained. The value of δ was set to 0.05. The compression set was constructed by searching for the smallest subset of training examples in which each k-mer in the model could be found. This problem is known as the minimum set cover problem and is NP-hard [3]. We therefore used the well-known greedy algorithm to approximate the solution to this problem [3], which yielded a small, but possibly not minimal, set of examples in which each k-mer in h could be found. The value of $m_{\mathcal{Z}}$ corresponds to the number of examples in this set. For simplicity, we assumed that each genome in the dataset had a size of 10 000 000, which is greater than the number of base pairs in any genome that we consider. Hence, $|\mathcal{Z}| = m_{\mathcal{Z}} \cdot 10\,000\,000$, which upper bounds the exact value of $|\mathcal{Z}|$. Notice that this approximation does not change the bound significantly since the bound only depends on the logarithm of $|\mathcal{Z}|$.

We compared the results obtained by selecting the hyperparameter values with both approaches. For cross-validation, we used $c = 5$ folds. The same experimental protocol as in Section *Results – The SCM models are sparse and accurate* of the main manuscript was

used to estimate the generalization performance of each approach. The results are shown in Table A3. In terms of average error rate on the testing set, a Wilcoxon signed-rank test [11] revealed that both methods perform comparably ($p = 0.463$). This indicates that our bound can indeed be used as a replacement for cross-validation. Moreover, these results suggest that our bound is sufficiently tight to provide insightful information on the generalization performance of the algorithm. Interestingly, the models obtained by using the bound instead of cross-validation were significantly sparser ($p = 0.014$), which is desirable in the context of biomarker discovery. In addition, using the bound instead of 5-fold cross-validation led to a sixfold decrease in the running time of the experiments. Finally, the small bound values obtained for some datasets highlight the tightness of our sample-compression bound. For example, the average bound value for the Benzylpenicillin models is 0.142. A bound value of 14.2% indicates that with probability 95%, any model learned by the SCM, from this dataset, will make no more than 14.2% predictions errors on examples drawn according to the same distribution D as the one that generated the training set $\mathcal{S}$.

Hence, we have provided empirical evidence that our sample-compression bound can be used as a replacement for c-fold cross-validation in the challenging setting of genomic biomarker discovery. This leads to sparser models with comparable accuracies and leads to a important reduction in the running time of the experiments.

Table A3: Comparison of cross-validation and the sample-compression bound (bound selection) as a hyperparameters selection strategy for the SCM. The values in the table are averages over 10 repetitions of the experiment. For each dataset and method, the error rate and the number of k-mers in the model (in parentheses) are shown. The average bound value obtained with the hyperparameters selected by bound selection is also shown (bound value). The best error rates are in bold.

Dataset	Cross-validation	Bound Selection	Bound Value
C. difficile			
Azithromycin	0.030 (3.3)	**0.025** (2.6)	0.251
Ceftriaxone	**0.073** (2.6)	0.089 (1.4)	0.379
Clarithromycin	**0.011** (3.0)	0.026 (2.6)	0.256
Clindamycin	0.021 (1.4)	**0.018** (1.8)	0.181
Moxifloxacin	**0.020** (1.0)	**0.020** (1.0)	0.174
M. tuberculosis			
Ethambutol	0.179 (1.4)	**0.172** (1.1)	0.564
Isoniazid	**0.021** (1.0)	**0.021** (1.0)	0.327
Pyrazinamide	**0.318** (3.1)	0.392 (1.4)	0.695
Rifampicin	0.031 (1.4)	**0.027** (1.0)	0.358
Streptomycin	**0.050** (1.0)	**0.050** (1.0)	0.393
P. aeruginosa			
Amikacin	**0.175** (4.9)	0.177 (2.7)	0.493
Doripenem	**0.270** (1.4)	0.275 (1.3)	0.562
Levofloxacin	**0.072** (1.2)	**0.072** (1.0)	0.324
Meropenem	**0.267** (1.6)	0.284 (1.7)	0.596
S. pneumoniae			
Benzylpenicillin	**0.013** (1.1)	0.011 (1.0)	0.142
Erythromycin	0.037 (2.0)	**0.035** (2.0)	0.210
Tetracycline	0.031 (1.1)	**0.027** (1.0)	0.164

Appendix 5 An efficient implementation

The large size of genomic datasets tends to surpass the memory resources of modern computers. Hence, there is a need for algorithms that can process such datasets without solely relying on the computer's memory. *Out-of-core* algorithms achieve this by making efficient use of external storage, such as file systems. Along with this work, we propose *Kover*, an out-of-core implementation of the Set Covering Machine tailored for presence/absence rules of k-mers.

This paragraph employs the same notation as in Section *Methods – Applying the Set Covering Machine to Genomes* of the main manuscript. Let $\mathbf{\Phi}$ be a $m \times |\mathcal{K}|$ binary-valued matrix, where each row corresponds to the $\boldsymbol{\phi}(\mathbf{x})$ vector of a learning example. A disk-based version of such a matrix can be obtained with the Ray Surveyor tool included in the Ray de novo assembler [12, 13]. This matrix contains all the information required to compute the utility function of the Set Covering Machine (Equation (3)). Indeed, let a_j and p_j be an absence and a presence rule for the j-th k-mer. Recall that, at each iteration of Algorithm 1, some examples are removed. Let $\mathcal{I}_\mathcal{P}$ and $\mathcal{I}_\mathcal{N}$ be the sets containing the indices of the remaining positive and negative examples. Then observe that, for the absence rule a_j, the terms of the utility function are given by:

$$|\mathcal{A}_{a_j}| = \sum_{i \in \mathcal{I}_\mathcal{N}} \mathbf{\Phi}_{i,j} \tag{6}$$

and that

$$|\mathcal{B}_{a_j}| = \sum_{i \in \mathcal{I}_\mathcal{P}} \mathbf{\Phi}_{i,j}. \tag{7}$$

Similarly, for the presence rule p_j, these terms are given by $|\mathcal{A}_{p_j}| = |\mathcal{I}_\mathcal{N}| - |\mathcal{A}_{a_j}|$ and $|\mathcal{B}_{p_j}| = |\mathcal{I}_\mathcal{P}| - |\mathcal{B}_{a_j}|$. Therefore, computing the utility function efficiently amounts to finding an efficient way of counting the non-zero entries in the binary matrix $\mathbf{\Phi}$.

Unfortunately, working with a binary matrix is inefficient, since in most modern operating systems, the minimum allowable unit of memory is the byte (8-bit). Therefore, to make efficient use of the memory resources, we pack the values of each column into 64-bit integers–thus obtaining an eightfold decrease in the amount of memory required to store the $\mathbf{\Phi}$ matrix. Moreover, we exploit the *popcount* atomic CPU instruction, which returns the number of bits that are equal to 1 in an integer, to efficiently compute the terms of the utility function. This leads to a tremendous speedup of up to 64 times in the computation of the utility function.

The key to an out-of-core implementation for the SCM is in computing the utility values without considering the entire $\mathbf{\Phi}$ matrix at once. To achieve this, we use HDF5 [14] to store the packed $\mathbf{\Phi}$ matrix on disk. This allows the matrix to be loaded in blocks during the computation of the utility function and therefore, it is never entirely stored in the computer's memory. Moreover, this library has a built-in compression scheme that further reduces the disk space required to store the data.

In summary, Kover, an out-of-core implementation of the SCM, is specifically tailored for genomic datasets. Kover is implemented in the Python and C programming languages, is open-source software and can be obtained from http://github.com/aldro61/kover.

References

[1] Marchand M, Shawe-Taylor J (2002) The set covering machine. *The Journal of Machine Learning Research* 3:723–746.

[2] Haussler D (1988) Quantifying inductive bias: AI learning algorithms and Valiant's learning framework. *Artificial intelligence* 36(2):177–221.

[3] Chvátal V (1979) A Greedy Heuristic for the Set-Covering Problem. *Mathematics of Operations Research* 4(3):233–235.

[4] Germain P et al. (2012) *A pseudo-boolean set covering machine.* (Springer), pp. 916–924.

[5] Pearson WR, Lipman DJ (1988) Improved tools for biological sequence comparison. *Proceedings of the National Academy of Sciences* 85(8):2444–2448.

[6] Hastie T, Tibshirani R, Friedman J (2013) *The Elements of Statistical Learning*, Data Mining, Inference, and Prediction. (Springer Science & Business Media).

[7] Littlestone N, Warmuth M (1986) Relating data compression and learnability, (University of California Santa Cruz, Santa Cruz, CA), Technical report.

[8] Floyd S, Warmuth M (1995) Sample compression, learnability, and the Vapnik-Chervonenkis dimension. *Machine Learning* 21(3):269–304.

[9] Marchand M, Sokolova M (2005) Learning with Decision Lists of Data-Dependent Features. *Journal of Machine Learning Research* pp. 427–451.

[10] Blumer A, Ehrenfeucht A, Haussler D, Warmuth M (1987) Occam's razor. *Information Processing Letters* 24:377–380.

[11] Wilcoxon F (1945) Individual Comparisons by Ranking Methods. *Biometrics Bulletin* 1(6):80.

[12] Boisvert S, Laviolette F, Corbeil J (2010) Ray: simultaneous assembly of reads from a mix of high-throughput sequencing technologies. *Journal of Computational Biology* 17(11):1519–1533.

[13] Boisvert S, Raymond F, Godzaridis É, Laviolette F, Corbeil J (2012) Ray Meta: scalable de novo metagenome assembly and profiling. *Genome biology* 13(12):R122.

[14] The HDF Group (1997-2015) Hierarchical Data Format, version 5. http://www.hdfgroup.org/HDF5/.
