# Supplementary material for: Predictive computational phenotyping and biomarker discovery using reference-free genome comparisons
Source: BMC Genomics. 2016 Sep 26;17:754. doi: 10.1186/s12864-016-2889-6 (PMC5037627; doi:10.1186/s12864-016-2889-6)
Supplement: Additional file 3 — Supplemental Tables. Table S1. A detailed overview of the sequencing data used in this study; Table S2. A detailed overview of the antibiotic resistance datasets used in this study; Table S3. An analysis of overfitting for the Set Covering Machine and support vector machines with linear and polynomial kernels; Table S4. Average and standard deviation of the sensitivities measured on the testing set for 10 random partitions of each dataset; Table S5. Average and standard deviation of the specificities measured on the testing set for 10 random partitions of each dataset; Table S6. Average and standard deviation of the error rates measured on the testing set for 10 random partitions of each dataset. (PDF 457 kb) [file 12864_2016_2889_MOESM3_ESM.pdf]

Table S1: A detailed overview of the sequencing data used in this study. For each species, the study accession number, sequencing platforms, number of genomes and a measure of sequence quality (FastQC) are shown.

Species	Accession	Sequencing Platform	Genomes	FastQC[1]
C. difficile	PRJEB11776	Illumina HiSeq 2500	470	98.1%
M. tuberculosis	PRJEB7281	Illumina MiSeq / HiSeq 2500	154	31.8%
P. aeruginosa	PRJNA264310	Illumina MiSeq / HiSeq 2000	390	64.6%
S. pneumoniae	PRJEB2632	Illumina HiSeq 2000	681	71.8%

[1] Proportion of the genomes that passed the FastQC quality control for per base sequence quality.

Table S2: The antibiotic resistance datasets used in this study. For each dataset, the number of genomes and k-mers, the type of antibiotic susceptiblity data (SIR[1] or MIC[2]) and any threshold used to separate the isolates into resistant and sensitive groups are shown. The resistant group (R) was assigned the positive class ($y = 1$) and the sensitive group (S) was assigned the negative class ($y = 0$).

	Genomes		Antibiotic Susceptibility			
Dataset	Examples	k-mers	Type	S	R	Threshold Source
C. difficile						
Azithromycin	462	32 752 570	MIC	≤ 8	> 8	Inferred[3]
Ceftriaxone	285	25 405 987	SIR	–	–	–
Clarithromycin	462	32 752 570	MIC	≤ 4	> 4	Bourgault et al. (2006)[4]
Moxifloxacin	462	32 752 570	MIC	≤ 4	> 4	Bourgault et al. (2006)[4]
M. tuberculosis						
Ethambutol	139	9 465 489	SIR	–	–	–
Isoniazid	141	9 701 935	SIR	–	–	–
Pyrazinamide	111	8 058 479	SIR	–	–	–
Rifampicin	141	9 701 935	SIR	–	–	–
Streptomycin	135	9 282 080	SIR	–	–	–
P. aeruginosa						
Amikacin	365	116 441 834	SIR	–	–	–
Doripenem	363	122 438 059	SIR	–	–	–
Levofloxacin	358	122 216 859	SIR	–	–	–
Meropenem	368	123 466 989	SIR	–	–	–
S. pneumoniae						
Benzylpenicillin	421	8 968 176	MIC	≤ 0.064	> 2	EUCAST[5]
Erythromycin	556	9 666 898	MIC	≤ 0.25	> 0.5	EUCAST[5]
Tetracycline	324	8 657 259	MIC	≤ 1	> 2	EUCAST[5]

[1] Sensitive/Intermediate/Resistant labels that were predefined by the authors of the dataset.

[2] Measures of minimum inhibitory concentration. In this case, a threshold was used to assign SIR labels to the isolates.

[3] No official MIC breakpoints were available. A visual inspection of the MIC distribution showed a clear separation between two groups of strains. The group with the lowest MIC values was deemed sensitive and the other one was deemed resistant.

[4] Bourgault, A. M., Lamothe, F., Loo, V. G., & Poirier, L. (2006). In vitro susceptibility of Clostridium difficile clinical isolates from a multi-institutional outbreak in Southern Quebec, Canada. Antimicrobial agents and chemotherapy, 50(10), 3473-3475.

[5] The European Committee on Antimicrobial Susceptibility Testing. Breakpoint tables for interpretation of MICs and zone diameters. Version 5.0, 2015.

Table S3: Analysis of overfitting: The training and testing[1] set error rates are shown for the set covering machine (SCM) and support vector machines with linear and polynomial kernels (LinSVM, PolySVM).

Dataset	SCM[2]	LinSVM[3]	PolySVM[3]
C. difficile			
Azithromycin	0.014 (0.030)	0.004 (0.050)	0.002 (0.048)
Ceftriaxone	0.042 (0.073)	0.008 (0.079)	0.010 (0.076)
Clarithromycin	0.017 (0.011)	0.003 (0.053)	0.002 (0.053)
Clindamycin	0.010 (0.021)	0.001 (0.039)	0.002 (0.039)
Moxifloxacin	0.019 (0.020)	0.028 (0.054)	0.029 (0.048)
M. tuberculosis			
Ethambutol	0.109 (0.179)	0.002 (0.215)	0.015 (0.221)
Isoniazid	0.014 (0.021)	0.004 (0.117)	0.011 (0.119)
Pyrazinamide	0.114 (0.318)	0.089 (0.382)	0.141 (0.382)
Rifampicin	0.013 (0.031)	0.000 (0.200)	0.001 (0.204)
Streptomycin	0.033 (0.050)	0.009 (0.143)	0.011 (0.148)
P. aeruginosa			
Amikacin	0.074 (0.175)	0.003 (0.184)	0.003 (0.179)
Doripenem	0.207 (0.270)	0.066 (0.288)	0.049 (0.281)
Levofloxacin	0.058 (0.072)	0.032 (0.221)	0.055 (0.225)
Meropenem	0.226 (0.267)	0.052 (0.329)	0.047 (0.331)
S. pneumoniae			
Benzylpenicillin	0.008 (0.013)	0.000 (0.015)	0.000 (0.015)
Erythromycin	0.021 (0.037)	0.004 (0.046)	0.004 (0.047)
Tetracycline	0.010 (0.031)	0.004 (0.039)	0.007 (0.037)

[1] The testing set error rate rates are shown in parentheses.

[2] SCM: the training and testing set error rates are generally similar, indicating that the SVM does not overfit.

[3] LinSVM and PolySVM: the training set error rates are generally much smaller than the testing set error rates. This indicates that the models are overfit to the training data.

Table S4: Average and standard deviation of the sensitivities measured on the testing set for 10 random partitions of the data. Results are shown for the set covering machine (SCM), the CART algorithm (CART), L_1 and L_2 regularized support vector machines (L1SVM, L2SVM), support vector machines with linear and polynomial kernels (LinSVM, PolySVM) and the baseline, which predicts the most abundant class in the training set. The prefix χ^2 indicates that univariate filtering was used.

Dataset	SCM	$\chi^2 + \text{CART}$	$\chi^2 + \text{L1SVM}$	$\chi^2 + \text{L2SVM}$	LinSVM	PolySVM	Baseline
C. difficile							
Azithromycin	0.967 ± 0.046	0.863 ± 0.068	0.923 ± 0.054	0.930 ± 0.055	0.939 ± 0.056	0.937 ± 0.051	0.000 ± 0.000
Ceftriaxone	0.940 ± 0.038	0.916 ± 0.051	0.931 ± 0.048	0.936 ± 0.050	0.956 ± 0.018	0.956 ± 0.018	1.000 ± 0.000
Clarithromycin	0.990 ± 0.009	0.881 ± 0.047	0.907 ± 0.058	0.918 ± 0.047	0.936 ± 0.029	0.930 ± 0.031	0.000 ± 0.000
Clindamycin	0.872 ± 0.089	0.954 ± 0.042	0.966 ± 0.036	0.886 ± 0.073	0.850 ± 0.085	0.850 ± 0.085	0.000 ± 0.000
Moxifloxacin	0.952 ± 0.023	0.952 ± 0.023	0.952 ± 0.023	0.920 ± 0.034	0.921 ± 0.031	0.918 ± 0.031	0.000 ± 0.000
M. tuberculosis							
Ethambutol	0.650 ± 0.200	0.661 ± 0.133	0.779 ± 0.165	0.476 ± 0.106	0.572 ± 0.101	0.560 ± 0.108	0.000 ± 0.000
Isoniazid	0.974 ± 0.018	0.974 ± 0.018	0.969 ± 0.025	0.856 ± 0.064	0.829 ± 0.060	0.832 ± 0.071	1.000 ± 0.000
Pyrazinamide	0.526 ± 0.308	0.536 ± 0.254	0.465 ± 0.305	0.388 ± 0.234	0.481 ± 0.251	0.513 ± 0.254	0.000 ± 0.000
Rifampicin	0.962 ± 0.028	0.957 ± 0.038	0.954 ± 0.043	0.663 ± 0.112	0.732 ± 0.080	0.725 ± 0.088	0.000 ± 0.000
Streptomycin	0.965 ± 0.052	0.966 ± 0.033	0.960 ± 0.050	0.844 ± 0.068	0.826 ± 0.100	0.822 ± 0.102	1.000 ± 0.000
P. aeruginosa							
Amikacin	0.467 ± 0.165	0.544 ± 0.090	0.517 ± 0.116	0.579 ± 0.092	0.495 ± 0.060	0.486 ± 0.064	0.000 ± 0.000
Doripenem	0.614 ± 0.161	0.615 ± 0.081	0.644 ± 0.061	0.581 ± 0.078	0.536 ± 0.116	0.483 ± 0.140	0.000 ± 0.000
Levofloxacin	0.890 ± 0.033	0.879 ± 0.030	0.859 ± 0.040	0.738 ± 0.038	0.727 ± 0.037	0.704 ± 0.067	0.000 ± 0.000
Meropenem	0.633 ± 0.063	0.663 ± 0.061	0.575 ± 0.061	0.568 ± 0.057	0.509 ± 0.090	0.517 ± 0.073	0.000 ± 0.000
S. pneumoniae							
Benzylpenicillin	0.873 ± 0.116	0.922 ± 0.077	0.927 ± 0.063	0.836 ± 0.119	0.806 ± 0.156	0.806 ± 0.156	0.000 ± 0.000
Erythromycin	0.891 ± 0.034	0.806 ± 0.075	0.879 ± 0.046	0.885 ± 0.035	0.834 ± 0.049	0.824 ± 0.060	0.000 ± 0.000
Tetracycline	0.875 ± 0.096	0.871 ± 0.078	0.875 ± 0.096	0.829 ± 0.112	0.779 ± 0.115	0.796 ± 0.109	0.000 ± 0.000

Table S5: Average and standard deviation of the specificities measured on the testing set for 10 random partitions of the data. Results are shown for the set covering machine (SCM), the CART algorithm (CART), L_1 and L_2 regularized support vector machines (L1SVM, L2SVM), support vector machines with linear and polynomial kernels (LinSVM, PolySVM) and the baseline, which predicts the most abundant class in the training set. The prefix χ^2 indicates that univariate filtering was used.

Dataset	SCM	$\chi^2 +$ CART	$\chi^2 +$ L1SVM	$\chi^2 +$ L2SVM	LinSVM	PolySVM	Baseline
C. difficile							
Azithromycin	0.973 ± 0.021	0.954 ± 0.032	0.947 ± 0.029	0.955 ± 0.029	0.959 ± 0.022	0.964 ± 0.025	1.000 ± 0.000
Ceftriaxone	0.904 ± 0.093	0.812 ± 0.127	0.874 ± 0.077	0.816 ± 0.060	0.845 ± 0.067	0.855 ± 0.066	0.000 ± 0.000
Clarithromycin	0.989 ± 0.012	0.969 ± 0.031	0.963 ± 0.022	0.959 ± 0.019	0.956 ± 0.019	0.961 ± 0.019	1.000 ± 0.000
Clindamycin	0.995 ± 0.009	0.994 ± 0.012	0.994 ± 0.015	0.993 ± 0.009	0.978 ± 0.017	0.978 ± 0.017	1.000 ± 0.000
Moxifloxacin	0.998 ± 0.004	0.998 ± 0.004	0.998 ± 0.004	0.971 ± 0.023	0.961 ± 0.026	0.973 ± 0.025	1.000 ± 0.000
M. tuberculosis							
Ethambutol	0.918 ± 0.081	0.901 ± 0.098	0.880 ± 0.069	0.951 ± 0.060	0.909 ± 0.052	0.906 ± 0.054	1.000 ± 0.000
Isoniazid	0.985 ± 0.024	0.984 ± 0.035	1.000 ± 0.000	0.896 ± 0.062	0.957 ± 0.040	0.953 ± 0.044	0.000 ± 0.000
Pyrazinamide	0.776 ± 0.150	0.704 ± 0.134	0.775 ± 0.142	0.818 ± 0.140	0.706 ± 0.267	0.694 ± 0.267	1.000 ± 0.000
Rifampicin	0.977 ± 0.026	0.980 ± 0.026	0.985 ± 0.025	0.925 ± 0.067	0.862 ± 0.049	0.858 ± 0.050	1.000 ± 0.000
Streptomycin	0.933 ± 0.048	0.928 ± 0.054	0.952 ± 0.048	0.881 ± 0.076	0.892 ± 0.078	0.887 ± 0.082	0.000 ± 0.000
P. aeruginosa							
Amikacin	0.928 ± 0.048	0.866 ± 0.056	0.900 ± 0.032	0.911 ± 0.041	0.906 ± 0.038	0.916 ± 0.034	1.000 ± 0.000
Doripenem	0.800 ± 0.069	0.809 ± 0.050	0.793 ± 0.033	0.806 ± 0.035	0.813 ± 0.047	0.847 ± 0.054	1.000 ± 0.000
Levofloxacin	0.960 ± 0.030	0.963 ± 0.028	0.963 ± 0.033	0.829 ± 0.027	0.822 ± 0.037	0.835 ± 0.042	1.000 ± 0.000
Meropenem	0.800 ± 0.041	0.790 ± 0.044	0.740 ± 0.068	0.746 ± 0.064	0.783 ± 0.059	0.775 ± 0.053	1.000 ± 0.000
S. pneumoniae							
Benzylpenicillin	0.997 ± 0.005	0.994 ± 0.010	0.994 ± 0.006	1.000 ± 0.000	1.000 ± 0.000	1.000 ± 0.000	1.000 ± 0.000
Erythromycin	0.976 ± 0.010	0.978 ± 0.010	0.972 ± 0.009	0.971 ± 0.013	0.974 ± 0.011	0.974 ± 0.011	1.000 ± 0.000
Tetracycline	0.982 ± 0.010	0.982 ± 0.010	0.980 ± 0.010	0.980 ± 0.010	0.983 ± 0.012	0.984 ± 0.010	1.000 ± 0.000

Table S6: Average and standard deviation of the error rates measured on the testing set for 10 random partitions of the data. Results are shown for the set covering machine (SCM), the CART algorithm (CART), L_1 and L_2 regularized support vector machines (L1SVM, L2SVM), support vector machines with linear and polynomial kernels (LinSVM, PolySVM) and the baseline, which predicts the most abundant class in the training set. The prefix χ^2 indicates that univariate filtering was used.

Dataset	SCM	χ^2 + CART	χ^2 + L1SVM	χ^2 + L2SVM	LinSVM	PolySVM	Baseline
C. difficile							
Azithromycin	0.030 ± 0.023	0.086 ± 0.020	0.064 ± 0.029	0.056 ± 0.026	0.050 ± 0.025	0.048 ± 0.023	0.446 ± 0.027
Ceftriaxone	0.073 ± 0.035	0.117 ± 0.032	0.087 ± 0.028	0.102 ± 0.033	0.079 ± 0.028	0.076 ± 0.028	0.306 ± 0.031
Clarithromycin	0.011 ± 0.006	0.070 ± 0.011	0.062 ± 0.020	0.059 ± 0.020	0.053 ± 0.013	0.053 ± 0.013	0.446 ± 0.027
Clindamycin	0.021 ± 0.010	0.011 ± 0.008	0.009 ± 0.011	0.021 ± 0.013	0.039 ± 0.010	0.039 ± 0.010	0.136 ± 0.026
Moxifloxacin	0.020 ± 0.008	0.020 ± 0.008	0.020 ± 0.008	0.048 ± 0.013	0.054 ± 0.019	0.048 ± 0.015	0.390 ± 0.024
M. tuberculosis							
Ethambutol	0.179 ± 0.056	0.185 ± 0.034	0.153 ± 0.045	0.221 ± 0.057	0.215 ± 0.043	0.221 ± 0.045	0.351 ± 0.071
Isoniazid	0.021 ± 0.016	0.021 ± 0.013	0.017 ± 0.012	0.125 ± 0.038	0.117 ± 0.033	0.119 ± 0.032	0.421 ± 0.064
Pyrazinamide	0.318 ± 0.104	0.371 ± 0.073	0.353 ± 0.066	0.342 ± 0.067	0.382 ± 0.108	0.382 ± 0.105	0.347 ± 0.065
Rifampicin	0.031 ± 0.017	0.031 ± 0.021	0.031 ± 0.021	0.196 ± 0.052	0.200 ± 0.033	0.204 ± 0.033	0.452 ± 0.070
Streptomycin	0.050 ± 0.045	0.052 ± 0.029	0.043 ± 0.046	0.137 ± 0.040	0.143 ± 0.050	0.148 ± 0.054	0.435 ± 0.049
P. aeruginosa							
Amikacin	0.175 ± 0.032	0.206 ± 0.042	0.187 ± 0.030	0.164 ± 0.030	0.184 ± 0.017	0.179 ± 0.018	0.216 ± 0.033
Doripenem	0.270 ± 0.038	0.261 ± 0.039	0.261 ± 0.028	0.275 ± 0.034	0.288 ± 0.028	0.281 ± 0.022	0.359 ± 0.026
Levofloxacin	0.072 ± 0.017	0.076 ± 0.016	0.085 ± 0.019	0.212 ± 0.019	0.221 ± 0.016	0.225 ± 0.017	0.463 ± 0.028
Meropenem	0.267 ± 0.014	0.261 ± 0.022	0.328 ± 0.032	0.327 ± 0.042	0.329 ± 0.026	0.331 ± 0.022	0.404 ± 0.024
S. pneumoniae							
Benzylpenicillin	0.013 ± 0.009	0.012 ± 0.010	0.011 ± 0.009	0.013 ± 0.011	0.015 ± 0.013	0.015 ± 0.013	0.073 ± 0.016
Erythromycin	0.037 ± 0.009	0.047 ± 0.010	0.041 ± 0.011	0.042 ± 0.009	0.046 ± 0.008	0.047 ± 0.009	0.142 ± 0.023
Tetracycline	0.031 ± 0.015	0.029 ± 0.008	0.032 ± 0.018	0.037 ± 0.012	0.039 ± 0.011	0.037 ± 0.012	0.106 ± 0.033
