# Supplementary material for: Predictive computational phenotyping and biomarker discovery using reference-free genome comparisons
Source: BMC Genomics. 2016 Sep 26;17:754. doi: 10.1186/s12864-016-2889-6 (PMC5037627; doi:10.1186/s12864-016-2889-6)
Supplement: Additional file 4 — Supplemental Figures. Figure S1. An illustration of the resistance models generated for each antibiotic. (PDF 54 kb) [file 12864_2016_2889_MOESM4_ESM.pdf]

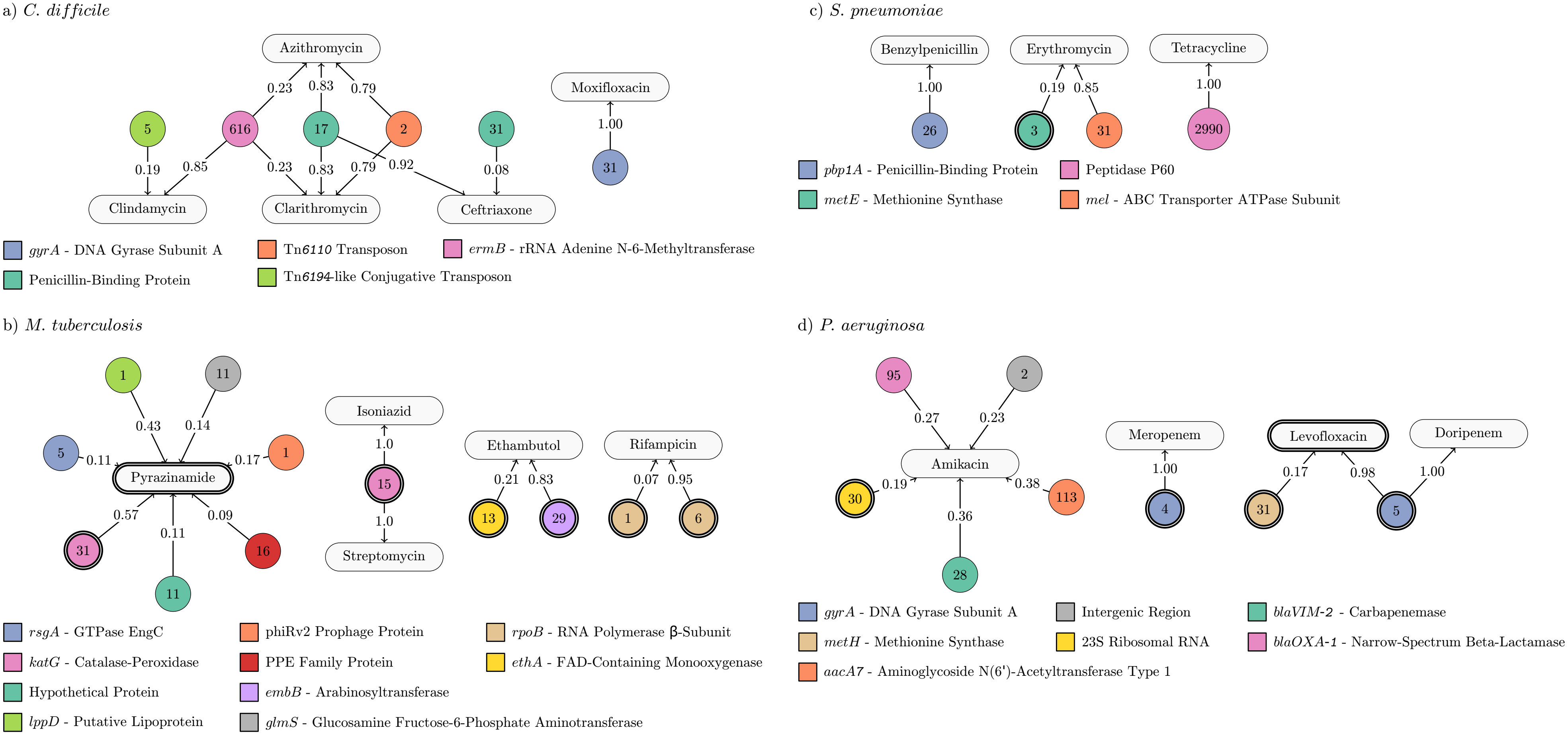

Figure S1: The antibiotic resistance models learned by the SCM for each dataset. The rounded rectangles correspond to antibiotics. A single border indicates that the model is a disjunction (logical-OR), whereas a double border indicates that the model is a conjunction (logical-AND). The circular nodes correspond to k-mer rules. A single border indicates a presence rule and a double border indicates an absence rule. The numbers in the circles show to the number of equivalent rules. A rule is connected to an antibiotic if it was included in its model. The weight of the edges gives the importance of each rule.
